# Relationship between redox activity and chemical speciation of size-fractionated particulate matter

**DOI:** 10.1186/1743-8977-4-5

**Published:** 2007-06-07

**Authors:** Leonidas Ntziachristos, John R Froines, Arthur K Cho, Constantinos Sioutas

**Affiliations:** 1 Department of Civil and Environmental Engineering, University of Southern California, Los Angeles, CA 90089, USA; 2 Center for Occupational and Environmental Health, School of Public Health, University of California Los Angeles, Los Angeles, CA 90095, USA; 3 Department of Molecular and Medical Pharmacology, School of Medicine, University of California Los Angeles, Los Angeles, CA 90095, USA

## Abstract

**Background:**

Although the mechanisms of airborne particulate matter (PM) related health effects remain incompletely understood, one emerging hypothesis is that these adverse effects derive from oxidative stress, initiated by the formation of reactive oxygen species (ROS) within affected cells. Typically, ROS are formed in cells through the reduction of oxygen by biological reducing agents, with the catalytic assistance of electron transfer enzymes and redox active chemical species such as redox active organic chemicals and metals. The purpose of this study was to relate the electron transfer ability, or redox activity, of the PM samples to their content in polycyclic aromatic hydrocarbons and various inorganic species. The redox activity of the samples has been shown to correlate with the induction of the stress protein, hemeoxygenase-1.

**Results:**

Size-fractionated (i.e. < 0.15; < 2.5 and 2.5 – 10 μm in diameter) ambient PM samples were collected from four different locations in the period from June 2003 to July 2005, and were chemically analyzed for elemental and organic carbon, ions, elements and trace metals and polycyclic aromatic hydrocarbons. The redox activity of the samples was evaluated by means of the dithiothreitol activity assay and was related to their chemical speciation by means of correlation analysis. Our analysis indicated a higher redox activity on a per PM mass basis for ultrafine (< 0.15 μm) particles compared to those of larger sizes. The PM redox activity was highly correlated with the organic carbon (OC) content of PM as well as the mass fractions of species such as polycyclic aromatic hydrocarbons (PAH), and selected metals.

**Conclusion:**

The results of this work demonstrate the utility of the dithiothreitol assay for quantitatively assessing the redox potential of airborne particulate matter from a wide range of sources. Studies to characterize the redox activity of PM from various sources throughout the Los Angeles basin are currently underway.

## Background

Epidemiological and toxicological studies have described associations between measured particulate matter (PM) mass and adverse health outcomes [1-4]. When considering plausible biological mechanisms of injury, PM mass may be a surrogate measure of other physical or chemical properties of PM that are the causal factors associated with the observed health outcomes. Several studies have since attempted to link health effects or toxicity measurements with particle characteristics such as particle size, number concentration and chemical composition. For example, there is accumulating evidence that ultrafine particles (with diameters less than about 100–150 nm) may be more toxic and biologically active on a per mass basis than larger particles [5,6]. Other studies have found associations with PM chemical constituents such as sulfate [7,8], trace elements and metals such as silicon [9], vanadium [10], iron, nickel and zinc [11], as well as elemental carbon [12,13], and polycyclic aromatic hydrocarbons (PAH) [14]. In general, results from these studies have been inconsistent due to the different health outcomes considered, the likelihood that health effects are induced by a combination of several physical or chemical properties of PM and the possibility of fortuitous associations, inherent in studies involving hundreds of measured organic and elemental chemical species that may be associated with the observed health effects.

Although the mechanisms of PM related health effects remain incompletely understood, an emerging hypothesis, currently under investigation, is that many of the adverse health effects derive from oxidative stress, of which one pathway is the formation of reactive oxygen species (ROS) within affected cells. There is a growing literature on health effects in association with cellular oxidative stress, including the ability of PM to induce pro-inflammatory effects in the nose, lung and cardiovascular system [5,15,16]. High levels of ROS cause a change in the redox status of the cell [17], i.e. the concentrations of the oxidized over the reduced species of cellular antioxidants such as glutathione [18], thereby triggering a cascade of events associated with inflammation and, at higher concentrations, apoptosis [19]. Typically, ROS are formed in cells through the reduction of oxygen by biological reducing agents such as NADH and NADPH, with the catalytic assistance of electron transfer enzymes and redox active chemical species such as redox active organic chemicals and metals [5,20].

PM has been shown to possess the ability to reduce oxygen to form ROS [21-23]. Li et al. [5] have reported a chemical assay involving the measurement of dithiothreitol (DTT) consumption that is capable of quantitatively determining superoxide radical anion formation as the first step in the generation of ROS. In this respect, the DTT assay measures a chemical property of the PM sample related to the ability if this sample to induce a stress protein in cells. Kuenzli et al. [24], measured the ability of ambient fine particles (≤ 2.5 μm) collected in various European cities to form hydroxyl radicals (•OH), as well as to deplete physiologic antioxidants (ascorbic acid, glutathione) in the reducing environment of respiratory tract lining fluid. The objective of their study was to examine how these toxicologically relevant measures were related to other PM characteristics. Correlations between oxidative activity and all other characteristics of PM were low, both within centers and across communities. Thus, no single surrogate measure of PM redox activity could be identified. Using a different bioassay than that of the Kuenzli et al [24] study, Chung et al [25] investigated the ability of PM-bound organic species such as quinones to generate reactive oxygen species (ROS) in PM samples collected in Fresno, CA, over a 12-month period. ROS generation was investigated by measuring the rate of hydrogen peroxide production from the reaction of laboratory standards and ambient samples with DTT. ROS generation from ambient samples in that study showed a strong positive correlation with the mass loadings of the three most reactive quinones and accounted for almost all of the ROS formed in the DTT test. In a previous study conducted by our group in a dynamometer emissions testing facility, Geller et al. [26] sought to determine the relationship between physical and chemical characteristics of PM and their redox activity in PM samples collected from diesel and gasoline passenger vehicles typically in use in Europe. Results from that study showed a high degree of correlation between several PM species, including elemental and organic carbon, low molecular weight PAHs, and trace metals such as nickel and zinc, and the redox activity of PM as measured by the DTT assay. The reduction in PM mass or number emission factors resulting from the various engine configurations, fuel types and-or aftertreatment technologies, however, was non-linearly related to the decrease in overall PM redox activity.

The present study is an extension of our efforts described by Geller et al. [26] and Cho et al. [22] to link PM characteristics from ambient samples to redox activity, using the DTT assay. It should be noted that the DTT assay is a chemical procedure conducted in buffer, not cell culture media. The purpose of the assay is to describe, in quantitative terms, the ability of the sample to transfer electrons from DTT to oxygen. Cellular studies with murine macrophages have shown a correlation of this activity with hemeoxygenase-1 induction ability [5], but no direct relationship to a health related endpoint such as asthma incidence has been shown for this or any other chemical assay. However, by using this assay and by conducting a new measurement campaign where PM samples were collected in various environments (road tunnel, freeway, background sites), the redox activity of different PM samples was determined and associated with their chemical characteristics. Also, a robust statistical analysis was conducted to underpin such associations. This field study was intended to contribute to the very limited body of literature linking PM characteristics to biologically meaningful properties such as the oxidative potential of atmospheric aerosols.

## Results

### Sample concentration and chemical speciation

**Figure 1:**
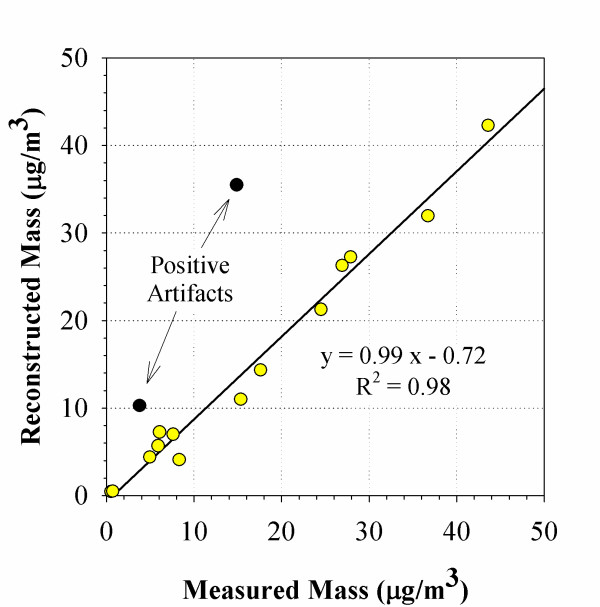
**Correlation between the gravimetrically determined and chemically reconstructed PM mass**. The figure shows a very good correlation between the PM mass determined gravimetrically from the Teflon filters and the PM mass reconstructed from chemical analysis (mostly on quartz filters) for samples collected in four different locations. There are two exceptions where positive adsorption artifacts are obvious for the quartz filter samples collected next to the CA-110 freeway. Excluding these two outliers, this graph indicates that the effect of these artifacts is limited at all other sampling locations.

The concentration and chemical speciation of PM samples collected in four different locations are shown in Table 1. The mass concentration of the PM samples is shown in the third column of the table. The remaining columns describe the results of the chemical analysis for EC and OC, nitrate, sulfate and inorganic metals and trace elements. The last column in this table shows the DTT activity (in nmoles of DTT consumed per min and per μg of PM), which is discussed in the following section. Organic carbon is the most abundant material in PM_2.5 _and PM_0.15 _modes in most of the samples. Organic carbon species may originate either directly from vehicle exhaust, which is a more prevalent PM source next to the CA-110 and in the Caldecott tunnel, or from secondary particle formation, which would be more pronounced in the receptor site of Riverside. However, organic material can be also collected due to adsorption of gaseous organic species on the filter surface, a process that results to a positive mass artifact on the filter. This is particularly true for the quartz filters used for the EC/OC analysis and it is the reason that the mass reconstruction by chemical analysis is higher than the weighted mass for three of the samples collected (Caldecott Bore 2- PM_0.15_, and CA-110 PM_0.15 _and PM_2.5_). Sampling artifacts (positive or negative) cannot thus be excluded for the other sampling locations. However, Figure 1 shows that with the exclusion of the two outliers from the CA-110 freeway, the mass concentration derived by filter weighing and the reconstructed PM mass from chemical analysis are in very good agreement, indicating that the effect of these artifacts is limited at all other sampling locations. The reconstructed mass varied between 79 and 95% of the measured mass, which confirms the consistency and overall reliability of the chemical measurements.

**Table 1:** Mass concentration, chemical composition and DTT activity of different PM size ranges in four sampling locations (nmol min^-1 ^μg^-1^).

Size Mode	Sampling Period	Location	Mass (μg m^-3^	EC (%)	OC (%)	NO3 (%)	SO4 (%)	Metals &Elements (%)	DTT activity (nmol min^-1 ^μg^-1^)
PM_0.15_	June 2003	Downey	5.0	5.0	41.0	6.5	16.0	20.4	0.061
	July 2003	Downey	5.9	2.0	41.0	4.9	17.6	31.1	0.083
	July 2003	Riverside	7.6	2.0	29.0	13.0	21.0	27.0	0.052
	August 2004	Riverside	7.6	3.7	43.8	17.1	9.1	6.7	0.053
	Sept. 2004	Caldecott B1	24.5	20.5	47.3	1.6	4.3	13.1	0.111
	Sept. 2004	Caldecott B2	0.6	10.5	74.7	1.9	4.9	27.7	0.172
	January 2005	CA-110	3.8	24.0	178.0	42.7	22.8	4.5	0.042

PM_2.5_	June 2003	Downey	17.6	2.0	18.0	24.0	6.5	31.0	0.036
	July 2003	Downey	43.6	1.0	32.0	21.0	7.0	36.0	0.021
	July 2003	Riverside	27.9	2.0	22.0	34.0	9.4	30.3	0.027
	August 2004	Riverside	26.9	2.0	22.0	34.0	9.4	30.3	0.028
	July 2005	Riverside	22.1	1.3	24.5	14.6	10.9	31.0	0.026
	Sept. 2004	Caldecott B1	36.7	4.8	48.9	3.3	3.3	26.8	0.068
	Sept. 2004	Caldecott B2	15.4	2.7	41.8	0.9	2.1	24.1	0.075
	January 2005	CA-110	14.9	25.8	148.0	39.7	15.3	9.9	0.025

Coarse	Sept. 2004	Caldecott B1	0.5	1.2	37.7	3.3	2.7	42.6	0.019
	Sept. 2004	Caldecott B2	0.7	0.4	14.0	0.6	2.2	52.8	0.032
	January 2005	CA-110	8.3	0.4	21.1	3.5	1.4	22.9	0.017

Elemental carbon is 10–25% of the PM_0.15 _mode next to the freeway and in the tunnel, while it represents a much lower fraction (< 5%) at background and receptor sites. This is a strong indication that EC in the Los Angeles Basin mainly originates from road traffic emissions. Nitrate (most of which is in the form of ammonium nitrate), is particularly high in Downey and Riverside compared to the rest of the locations. The high nitrate levels at Riverside are of particular note and consistent with previous studies in that area, and reflect the result of atmospheric reactions of nitric acid with fugitive ammonia, largely emitted from the nearby upwind dairy farms in the area of Chino, CA [27,28]. Sulfate concentrations are generally low and do not show any clear trend with proximity to freeways or to receptor sites, an indication that sulfate in this area is mostly associated with the regionally dispersed, spatially homogeneous background aerosol.

The inorganic and trace elements detected accounted for up to ~50% of the total mass for coarse particles. Their fraction decreased in PM_2.5 _and especially PM_0.15 _samples. The most abundant single element detected was Si, with a mass fraction ranging between 1–17% of PM mass. The second most abundant element was iron (Fe) with a fraction reaching up to 15% in the coarse mode. The abundance of Fe was up to 10% also in the PM_0.15 _mode, for samples collected in sites affected by traffic. Al, Ca and Cl were the third more abundant species, with relative abundances ranging from 0.5% (0.05% for Cl) to 10% of the PM mass in each size range. All other elements were found at lower concentrations. Na and S reached up to 3%, Mg, K, Ti, Cu, Zn and Ba reached up to 1% of the total PM mass per size mode. Finally, a number of other trace elements were detected, with a contribution at- or below 0.1% of the total mass. The most significant of those, with decreasing order of abundance (mean value for all samples shown in parentheses), were Sb (0.1%), Mn (0.1%), Pb (0.08%), Sn (0.05%), Zr (0.05%), P (0.04%), Cr (0.03%), Ni (0.04%) and V (0.03%).

**Figure 2:**
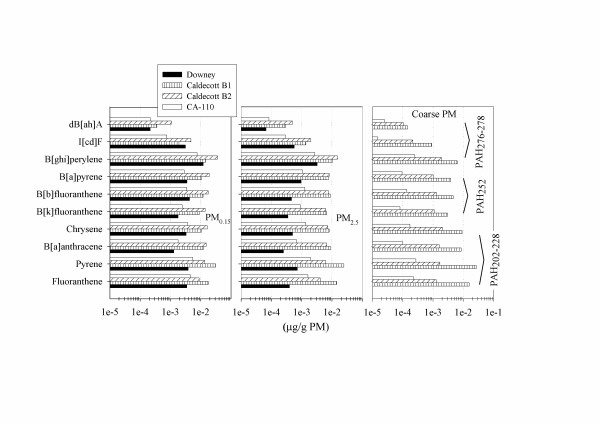
**PAH content in size-fractionated PM samples, per sampling location**. The PAHs have been grouped according to their molecular weight as schematically shown on the rightmost panel for all four sampling locations. Note the logarithmic scale on the x-axis. The highest PAH concentrations were found for samples collected in the tunnel. Interestingly, the concentration of heavier PAHs is higher in the gasoline-only tunnel bore while the PAH profile is shifted to relatively lighter components in the mixed gasoline-diesel bore.

PAH were also measured with the exception of samples collected in Riverside (Figure 2). The PAH species were divided into three groups for analysis, based on their molecular weight. The first group consisted of four species (Fluoranthene, Pyrene, Benz [a]anthracene and Chrysene) with a molecular weight between 202 and 228. The second group consisted of the PAHs (Benzo [k]fluoranthene, Benzo [b]fluoranthene, Benzo [a]pyrene) with a molecular weight of 252. The third group was comprised of PAHs with molecular weight in the range of 276–278 (Benzo [g, h , i]perylene, Indeno [1,2,3-cd]pyrene, Dibenz [a, h]anthracene). Generally, PAH concentrations increase with decreasing particle size, with the maximum concentrations observed for the two lighter species (Fluoranthene and Pyrene). The maximum total PAH concentrations were found for samples collected in the roadway tunnel. The concentration of heavier PAHs is higher in the gasoline-only tunnel, while the concentration of lighter components (PAH202-228) is higher in Bore 2, where diesel traffic is also permitted. This is consistent with previous studies [29,30] which showed that the PAH profile of gasoline vehicles is shifted towards the heavier molecular weight species compared to diesel vehicles.

### DTT activity

The DTT activities of the 18 samples are shown in the last column of Table 1. The DTT activity is highest in the PM_0.15 _mode, followed by the PM_2.5 _and the coarse modes at all sampling sites, with averages of 0.088 (± 0.040), 0.038 (± 0.022) and 0.023 (± 0.009) nmoles DTT per min per μg PM for ultrafine, PM_2.5 _and coarse PM, respectively. The PM_2.5 _fraction contains all PM less than 2.5, including the PM_0.15 _mode. Similar observations regarding the effect of particle size on per mass DTT activity were also made by Cho et al. [22] in their tests in various locations in the Los Angeles Basin. More importantly, the redox activity becomes maximum for PM_0.15 _sampled in the road tunnel, which is directly influenced by the on-road fresh emissions. Interestingly, the highest DTT activity per mass of PM was associated with the sample collected in the gasoline only tunnel bore (B2) of the Caldecott tunnel. This result is also consistent with the dynamometer study by Geller et al. [26], that showed higher PM redox activity per mass of PM emitted by a gasoline over a diesel vehicle.

## Discussion

### Univariate correlation between redox activity and PM species

As a first step in our exploratory data analysis, we attempted to identify correlations between the DTT redox activity measured in PM samples and their composition in EC, OC ions, elements and PAHs. Table 2 shows the Pearson correlation coefficients (*R*) and the associated coefficient of significance (*p*). All particle size ranges (PM_0.15_, PM_2.5 _and coarse PM) have been combined in this correlation. This analysis shows limited and not statistical significant correlation of DTT activity with EC, NO_3_, SO_4 _and the sum of metals and elements determined. None of the individual metals and elements measured was significantly correlated with DTT activity, with the exception of Cr, which led to a Pearson coefficient of 0.65 albeit not statistically significant at a significance level of 0.05. The correlation between DTT and OC levels was not significant when all 18 samples are taken into account. However, this includes the two unrealistically high OC values determined in the PM_0.15 _and PM_2.5 _samples next to the CA-110 (Table 1), a likely result of positive sampling artifacts, as discussed earlier. Exclusion of these two values leads to a much improved, and statistically significant correlation (R = 0.87). All measured PAHs were significantly correlated with DTT activity at the p = 0.05 level. Most importantly, DTT activity is highly correlated with the heavier classes of PAH species, with Pearson coefficients as high as 0.95 for the 13 samples for which PAH analysis was available. The difference between the correlation coefficient for the lighter PAHs and the heavier species may reflect differences in the volatility of the redox active species. Also, lighter PAHs may also be prone to sampling artifacts, similar to total OC. However, in our case, removal of the two CA-110 samples did not significantly change the correlation. Despite these differences, the results of Table 2 show that the organic component of the PM samples is an important factor in determining their redox activity. These correlations were established independently of particle size, i.e. they are applicable for the whole size range of inhalable particles.

**Table 2:** Pearson correlation coefficients (R) and level of significance (p) for DTT activity with different PM species

Species	R	p
EC	0.26	0.30
OC	0.12	0.64
OC (excluding two unrealistic values)	0.87*	< 0.01
NO_3_	-0.45	0.06
SO_4_	-0.08	0.75
Metals and elements	-0.19	0.45
PAH 202–228 (FLU, PYR, BaA, CHR)	0.57*	0.04
PAH 252 (BkF, BbF, BaP)	0.92*	< 0.01
PAH 276–278 (BghiP, IcdP, dBahA)	0.95*	< 0.01

* indicates significance at the p = 0.05 level

**Figure 3:**
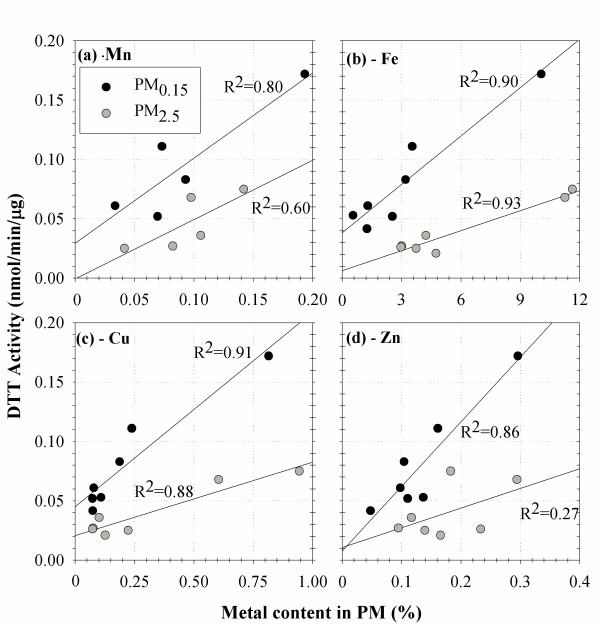
**Correlation of DTT activity with different transition metals**. Particulate samples are distinguished in two different particle size fractions (PM_0.15 _and PM_2.5_). The four panels show the correlation of the DTT activity of two different PM size fractions (PM_0.15 _and PM_2.5_) with (a) Mn, (b) Fe, (c) Cu, (d) Zn, respectively. These graphs show that despite the DTT activity of PM samples is not correlated with transition metals in the pooled samples of different size ranges (Table 2) there is a strong correlation with some metals within the two particle size fractions. This may probably be an artifact of the high correlation of metals and PAHs in these samples.

We further explored correlations of DTT activity with individual species within each particle size mode. Table 3 presents the results of these correlations for the PM_0.15 _and PM_2.5 _modes, for which an adequate sample size was available. The results in this table demonstrate that there is no correlation with EC and a significantly negative correlation at the p = 0.05 level with NO_3 _and SO_4_. None of these species can be considered responsible for the redox activity of the PM samples, even within each particle size mode. In contrast, the positive DTT correlation with OC is evident even within each particle size mode. Table 4 also shows high Pearson coefficients between the DTT activity and several transition metals, such as Mn, Fe, Cu, Zn, and metals Pb and Ba. The correlations with Mn and Zn are only significant in the PM_0.15 _mode. The DTT correlation with transition metals is also shown in Figure 3.

**Table 3:** Pearson correlation coefficients (R) and level of significance (p) for DTT activity of species measured within the PM_0.15 _and PM_2.5 _size ranges

Species	PM_0.15_		PM_2.5_	
	R	p	R	p
EC	0.14	0.77	-0.18	0.70
OC^a^	0.92*	0.01	0.79*	0.05
NO_3_	-0.63	0.13	-0.81*	0.03
SO4	-0.75*	0.05	-0.80*	0.03
Metals and elements	0.44	0.31	-0.12	0.80
Na	-0.66	0.11	0.03	0.95
Mg	-	-	-0.52	0.29
Al	-0.10	0.83	-0.67	0.10
Si	-0.04	0.93	-0.63	0.13
Cl	0.15	0.75	0.63	0.13
K	-0.06	0.89	-0.69	0.09
Ca	0.55	0.20	-0.62	0.14
Ti	0.66	0.11	0.67	0.10
V	0.32	0.53	0.19	0.76
Cr	0.53	0.28	0.86*	0.05
Mn	0.90*	0.01	0.78	0.12
Fe	0.95*	< 0.01	0.96*	< 0.01
Ni	0.55	0.26	-0.46	0.36
Cu	0.95*	< 0.01	0.94*	< 0.01
Zn	0.93*	< 0.01	0.52	0.23
Br	-0.30	0.52	-0.54	0.21
Sr	0.74	0.09	0.70	0.12
Zr	0.80	0.10	0.86	0.06
Sn	0.71	0.18	-0.10	0.87
Ba	0.89*	0.04	0.92*	0.01
Pb	0.95*	< 0.01	0.88*	0.02

^a ^Two unrealistic values have been removed from the OC samples, similar to Table 2.

* indicates significance at the p = 0.05 level

Although the redox activity of transition metals in biological reactions is well-established [31,32], the DTT assay in general does not reflect metal-based redox activity [22] especially for transition metals such as Cu and Fe. An important finding of the current study, however, is that while transition metals are not correlated with DTT activity in the pooled samples of particles from different size ranges, a strong correlation exists for PM_2.5 _and PM_0.15 _samples. As evidenced in the results shown in Table 3, several of the transition metals in the PM_0.15 _and PM_2.5 _ranges are highly correlated with PAH, possibly due to their common sources (e.g. vehicle exhaust emissions). For example, the Pearson correlation coefficients between PAH with molecular mass above 252 and Mn, Fe, Cu, and Zn are 0.86, 0.95, 0.96 and 0.99, respectively. Hence, the very high correlations of transition metals and DTT activity in Figure 3 might more probably be attributed to the high correlation of metals and PAH in the PM samples, especially in the PM_0.15 _mode.

### Multivariate correlations between PM chemical constituents and DTT activity

#### The role of PAHs

The previous analysis demonstrated that the redox activity of PM is highly correlated with their PAH content and, depending on PM size fraction, with their content in transition metals. The univariate regressions employed in the analysis of the previous section may not be in the position to discriminate and independently quantify the impact of PAHs and transition metals. We therefore applied multivariate regressions in order to separate their effects. This was performed by means of SPSS 12.0 on the samples for which PAH analysis was available. The results in Table 2 showed that PM redox activity is highly correlated to both medium and heavy PAH species, with Pearson coefficients exceeding 0.9 in both cases. We therefore decided to group these two PAH categories together and apply the regression to the sum of PAH species with molecular weight above 252. It is reminded that this pooled sample consists of six species (BkF, BbF BaP, BghiP, IcdP, dBahA).

**Figure 4:**
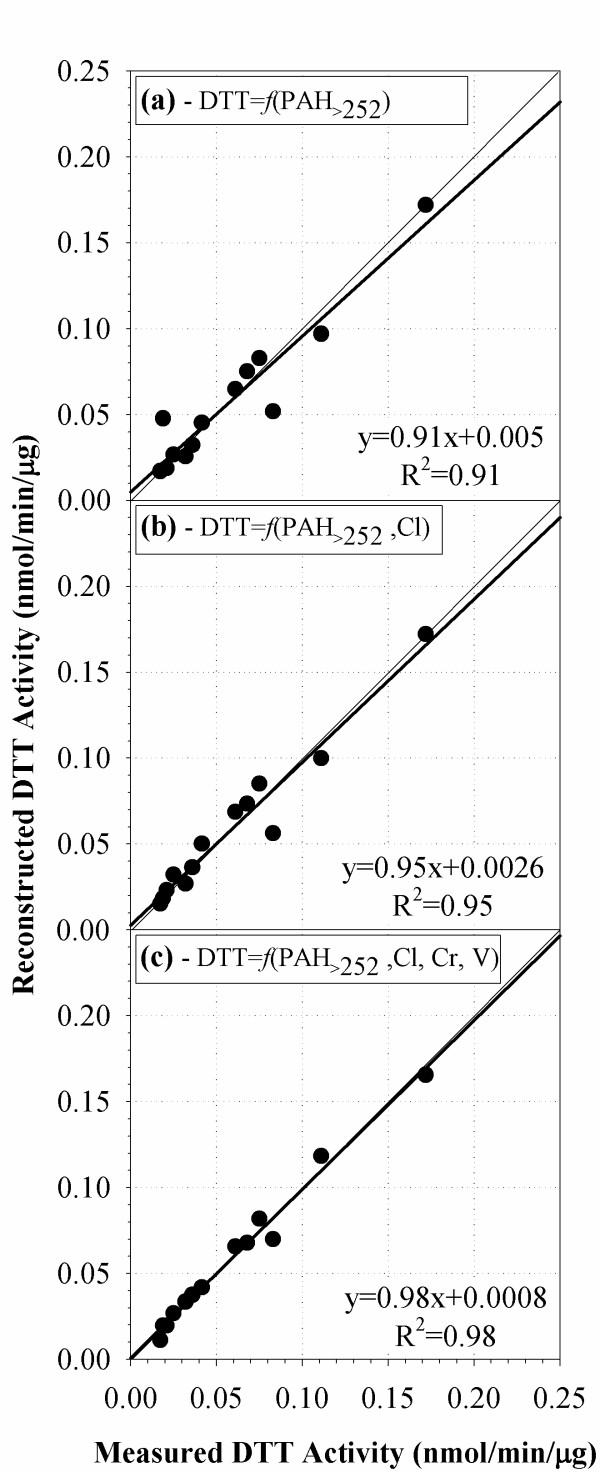
**Correlation of measured and reconstructed DTT activity for samples in all size modes**. (a) Correlation with PAH of molecular weight equal or greater than 252, (b) Correlation with PAH and Cl, (c) Correlation with PAH, Cl, Cr and V. The thin line is the chart diagonal representing the 1:1 line. The correlations demonstrate the strong correlation of DTT activity and PAH which may act as surrogates for other functional groups that have the capacity to reduce oxygen, such as quinones and aromatic nitro-PAHs. The correlation also improves and the intercept decreases when, gradually, Cl, Cr and V are introduced in the regression. The regression parameters that can be used to quantify the effects of each species on the DTT activity are given in Table 4.

The multivariate regression employed was performed in three steps. As a first step, the regression only involved the measured DTT activity and the total mass of these six PAH species. This regression yielded a high correlation coefficient (R^2^) of 0.91. The reconstructed DTT activity based on this correlation is plotted as a function of the measured DTT activity in Figure 4a. The slope of the correlation is 0.91 and the intercept is 0.005, which corresponds to 28% of the minimum DTT activity measured. This is already a very satisfactory correlation, especially considering that it includes samples in three different particle size modes, collected in four different locations (Downey, CA-110, and Caldecott Bore 1 and 2). It therefore indicates that the heavy PAH content of PM is a very robust indicator of their redox activity.

PAHs do not contain functional groups that have the capacity to reduce oxygen and form the superoxide radical anion. However, relevant oxygenated and-or other redox active functional groups constituents can be generated from the transformation of PAH via combustion, atmospheric chemistry or in vivo biotransformation. For example, Sun et al. [33] demonstrated the formation of two benzo(a)pyrene quinones (1,6 and 3,6-quinones) via biotransformation of the parent compound coated on diesel particles. There are numerous cites on the larger molecular weight PAHs for quinone formation, and therefore a particular PAH may lead to DTT activity that reflects a number of quinone isomers formed from the parent compound. Schuetzle et al. [34] reported a wide range of organic compounds generated by vehicles, including PAH-quinones, PAH ketones, and carboxaldehydes, all of which may be transformed to quinones via atmospheric chemistry or biotransformation. Other compounds that have potential DTT activity include aromatic nitro-PAH groups that are formed via atmospheric chemistry [35]. Schuetzle et al. [34] reported a number of nitro-PAHs from vehicle emissions. The emissions of nitro-PAHs from diesel vehicles have been described in a wide range of publications summarized by Arey [35]. These compounds have not been investigated in the DTT reaction to date.

#### Combined effect of PAHs and inorganic species in the statistical correlation

The second step in the multivariate regression, involved the introduction of each of the additional species measured (ions, elements and metals) in the regression. This was performed on one-by-one basis, by examining the statistical significance (significance level 0.05) of the improvement in the correlation, introduced by each species. Interestingly, the correlation of halogens (Cl, Br) with the DTT activity was found to be negative at a significance level of < 0.05 after the PAH effect was taken into account. However, only Cl was included in the final multivariate regression because Br was not measured for one of the samples and its inclusion would reduce the sample size. The restructured DTT activity, taking into account both PAH and Cl, over the measured one is shown in Figure 4b. By taking Cl into account, the correlation coefficient increased to 0.95 and the intercept further decreased to 0.0026 (15% of the minimum measured value).

The third step was similar to the second one, by examining species that could further improve the correlation on a statistically significant basis. The analysis indicated that both Cr and V could independently lead to an improvement in the correlation at a significance level of 0.05. We thus combined the mass fractions of these two metals per sample and applied the regression on their sum. The final reconstructed signal over the measured one is shown in Figure 4c. The resulting correlation coefficient is 0.98 and the intercept is almost zero. All species (variables) included in the regression are mutually independent, as revealed by their very low correlation coefficients (max R^2 ^of 0.22 between PAH and Cr). No additional species improved the correlation at a statistically significant level, hence the multivariate regression was concluded at this third step. As a result, no other transition metals appeared in the multivariate regression. This is probably because Mn, Fe, Cu, and Zn are already highly correlated with PAHs within each particle size range and thus offer no additional explanation of the DTT activity. On the other hand, Cr and V are independent of PAHs and their effect on PM redox characteristics becomes evident.

The presence of Cr and V in the regression model should probably be considered only an effect of their statistical independence to PAH. It is conceivable that more transition metals would appear in this correlation in a larger dataset of PM samples, possibly collected in more locations impacted by a variety of sources, where metals and PAHs would be independent. This finding illustrates perhaps one of the most serious limitations of any study attempting to link toxicological PM properties strictly to their chemical composition: The inevitable association between species originating from the same source (or group of sources) confounds our ability to assess the degree to which they are individually responsible for toxic effects attributable to PM. An alternative, and possibly more effective approach in determining PM toxicity is to link the toxic potential of PM to different sources by using source apportionment techniques based on particle chemical composition [36].

Recognizing the limitations discussed above, the regression parameters can be used to quantify the effect of each species on the DTT activity. Table 4 summarizes this information. The reconstructed DTT activity shown in Figure 4c is calculated as a summation of the products of the unstandardized coefficient values with the PAH sample content (in μg PAH per g of PM mass), Cl (%) and Cr+V (%). The last column in Table 4 shows the significance level for all independent variables utilized and confirms their statistical significance. It also illustrates the fact that a constant (intercept) is required to bring the reconstructed DTT activity at the same level with the measured one. To date, Cl, Cr and V have not been assayed individually for DTT activity, and their contribution may reflect a statistical artifact, but further investigations are necessary. In general, metals and ions are not active in the DTT assay, as noted earlier, because the DTT assay measures superoxide radical anion formation, and is not an element of the Fenton reaction, where metals serve as catalysts for hydroxyl radical formation.

**Table 4:** Parameters of the multivariate regression analysis between DTT activity and PM chemical composition

Independent Variables	Unstandardized Coefficients		Standardized Coefficients	Significance level
	Value	Std. Error		
Constant	0.0152	0.0033		0.001
PAH > 252(μg per g of PM mass)	1.43 × 10^-4^	8.78 × 10^-6^	0.812	< 0.001
Cl (%)	-3.40 × 10^-3^	7.3 × 10^-4^	-0.199	0.001
Cr+V (%)	0.166	0.0386	0.214	0.002

The relative contribution of each independent variable on the DTT activity can be obtained by the coefficients of the standardized variables. These coefficients correspond to the change of the standardized DTT activity variable per standard variation change of any of the independent variables. For example, the DTT activity would change by 0.81 standard deviations (0.035 nm min^-1 ^μg^-1^) for one standard deviation change of PAHs (243 μg per g of PM mass). On the other hand, the DTT activity would only change by 0.21 standard deviations per standard deviation change in Cr+V (0.071%) in the PM sample. If this is considered as an indicator of relative potency of each species, PAHs with a molecular weight above 252 have ~4 times higher redox inducing potency than the sum of Cr and V in the sample.

We need to emphasize again that it is premature at this stage to draw conclusions about the role of transition metals in the redox activity of PM when measured by the DTT assay. However, in addressing the toxicity of airborne PM, it is apparent that a wide range of organic compounds and metals may be actively involved in the resulting health effects through ROS formation and oxidative stress or direct electrophilic reactions. Some of these compounds will be active in the DTT reaction, e.g., naphthoquinones and phenanthroquinones, but others will exert their toxicity while interacting with macromolecules (enzymes and DNA) through direct chemical covalent bond formation. The result of electrophilic chemistry will be oxidative stress impairment via signal transduction pathways, which represents a broader, more complete definition of oxidative stress. This pathway has been demonstrated for both quinones and metals [37,38]. Examples include organic species such as benzoquinone, naphthoquinone and phenanthroquinone, and metals such as Zn. Therefore some species may act to elicit oxidative stress via two pathways, ROS formation and electrophilic chemistry. Future research should focus on the formation of ROS because of the catalytic nature of the process as well as the electrophilic chemistry that results in irreversible bond formation and subsequent toxicity.

## Conclusion

The results of this work, combined with the earlier findings by our center [5,22,26], demonstrate the utility of the DTT assays for quantitatively assessing the redox potential of airborne particulate matter from a wide range of sources. First, the DTT assay resulted in higher redox activity for PM samples in the ultrafine mode, while the activity decreased for PM samples in the fine and coarse modes respectively. Given that the assay should not be sensitive to the physical particle dimensions, the observed associations will have to be attributed to the distinct chemical character of particles in different size fractions. Second, the correlation of DTT activity with PAHs indicates that organic compounds with affinity to PAH or PAH derivatives are responsible for the redox properties of the PM samples. Finally, inorganic species such as metals, that may be actively involved in the resulting health effects through ROS formation and oxidative stress or direct electrophilic reactions, also show up in the correlations. Although these species are not expected to contribute to the measured DTT consumption rate by means of a direct chemical mechanism, their presence in the statistical associations demonstrates that they act as surrogates of a particularly redox active PM source.

Information collected by the DTT and similar assays would allow for more effective regulatory strategies with respect to pollution source control, more targeted air quality standards, and ultimately, reductions in population exposure to the most harmful types of PM. Furthermore, once the most health relevant PM sources are identified, the list of hazardous particle characteristics can be narrowed down, thereby making more targeted mechanistic investigations of PM health effects possible.

## Methods

### Sampling sites and periods

Sampling took place at four diverse sites, during the period of June 2003 to July 2005. These sites were at Downey, Riverside, the vicinity of the CA-110 freeway, and the two bores of the Caldecott tunnel. The first three sites are located in the Los Angeles basin, whereas the Caldecott tunnel is located in the metropolitan area of San Francisco, at Orinda, CA. Detailed information about the Los Angeles Basin sites is given by Sardar et al. [28]. Briefly, Downey, located in central Los Angeles, is downwind of the "Alameda corridor", a narrow industrial zone and transportation route between the Ports of Los Angeles/Long Beach and Downtown Los Angeles. The area is characterized by a high density of diesel trucks, which serves to transfer overseas cargo from the port to industrial sites, warehouses, and the rail yards near downtown Los Angeles. The Downey site is approximately 10 km downwind of some oil refineries, 1–2 km downwind of two major freeways, and is heavily impacted by vehicular sources. Riverside is about 90 km east of downtown Los Angeles. The site is also about 25 km downwind of the Chino area dairy farms, a strong ammonia source leading to high concentrations of ammonium nitrate [39]. The area is upwind of surrounding freeways and major roads. The predominantly westerly wind transports particles generated near central Los Angeles toward Riverside, resulting in an aged and photochemically processed aerosol. Riverside is also characterized by some of the highest PM levels in the Basin. Measurements at the CA-110 freeway were conducted at 2.5 m from the edge of the freeway (as described in Kuhn et al. [40]). This freeway connects downtown Los Angeles and Pasadena, CA. On this stretch of the freeway, only light-duty vehicles are permitted, thus affording a unique opportunity of studying emissions from pure light-duty traffic under ambient conditions. Finally, the 1.1-km long Caldecott Tunnel includes three two-lane bores with a 4.2% incline from west to east. Bores 1 and 3 allow both light-duty vehicles and heavy-duty vehicles, while Bore 2 is restricted to light-duty vehicle traffic only. Traffic flows from west-to-east in Bore 1, east-to-west in Bore 3, and the direction of traffic switches from westward in the morning to eastward in the afternoon and evening in Bore 2. Field sampling was conducted in the afternoon in Bores 1 and 2 (B1 and B2) for 4 days each from approximately 12 p.m. to 6 p.m., when all traffic in the two bores traveled eastward.

In each site, size fractionated PM samples were collected over a period of 2–3 weeks, for about 5 days per week, and 6–7 hrs/day. Thus each sample is a composite of some 70–100 hrs of collection.

### Sampling process and analysis

Coarse (i.e. particles with aerodynamic diameter 2.5–10 μm), fine (PM_2.5_, < 2.5 μm), and ultrafine (PM_0.15 _< 0.15 μm) particles were collected at the these sites by the Versatile Aerosol Concentration Enrichment System (VACES), in a process described in greater detail by Li et al. [5] and Cho et al. [22]. Briefly, the VACES uses three parallel sampling lines (concentrators) to simultaneously collect coarse and fine particles at a flow rate of 120 liters per minute (lpm) into a liquid impinger (BioSampler™, SKC West Inc., Fullerton, CA) at 5 lpm. Particles are injected into the BioSampler in a swirling flow pattern so that they can be collected by a combination of inertial and centrifugal forces. This inertia-based collection mechanism, coupled with the short residence time on the order of 0.2 s for particles and gases in the Biosampler precludes any inadvertent trapping of gaseous co-pollutants in the particulate layer.

In each sampling line of the concentrator, coarse PM, PM_2.5 _and PM_0.15 _were concentrated from a flow of 120 lpm to a flow of 6 lpm, thereby, being enriched by a factor of 20. From the 6 lpm of concentrated flows samples, 4 lpm was drawn through the BioSampler connected to the respective minor flow, while 2 lpm passed through diffusion dryer for PM_2.5 _and PM_0.15 _only to remove excess water and dry the aerosol. Diffusion drying of coarse PM was not considered necessary since it is concentrated without hydration of the aerosol. The dry concentrated aerosol flow was then split into two equal halves of 1 lpm, each diverted into a filter sampler consisting of either a 47-mm Teflon filter (2-mm pore PTFE; Gelman Science, Ann Arbor, MI) or a 47 mm prebaked quartz filter (Pallflex Corp., Putnam, CT). The PTFE filters were used to determine particle mass and the metal content, whereas quartz filters were used to determine the PM content of elemental and organic carbon (EC-OC), inorganic ions, and PAHs.

For measurement of mass concentrations, the PTFE filters were weighed before and after each field test using a Mettler 5 Microbalance (MT 5, Mettler-Toledo Inc., Highstown, NJ), under controlled relative humidity (40–45%) and temperature (22–24°C) conditions. At the end of each experiment, filters were stored in the control humidity and temperature room for 24 h prior to weighing to ensure removal of particle-bound water. The concentration of trace elements and metals was determined by means of X-ray fluorescence subsequent to filter weighing. The quartz filters were cut into two unequal parts, 1/4 and 3/4 of the total filter. The smaller piece was analyzed by means of ion chromatography to determine particle-bound sulfate and nitrate concentrations. A small area (1 cm^2^) of the remaining filter (3/4) was removed to determine the EC and OC content of PM. EC and OC were determined using a thermal optical transmittance method as specified in NIOSH method 5040 (Birch [41]). The remaining portion from the filter above was used to determine the concentrations of PAH using procedures described elsewhere [42]. In brief, the filters corresponding to each size range were ultrasonically extracted with dichloromethane and the PAH content of the dichloromethane extract was analyzed by high-pressure liquid chromatography fluorescence using NIST SRM 1649a as the positive control.

The DTT assay is described in greater detail by Cho et al. [22]. This assay provides a measure of the overall redox activity of the sample based on its ability to catalyze electron transfer between DTT and oxygen in a simple chemical system. We have used this assay because it provides a quantitative measure of redox activity that can be normalized to mass or air volume such that samples from different sources can be compared. Other assays used for this purpose include the consumption of ascorbate [43], oxidation of dichlorofluorescin [44] and an ESR procedure [45] to monitor the levels of free radical species. We have not compared the DTT assay directy to other assays, but have observed that ascorbate consumption by PM is sensitive to metal chelating agents, whereas DTT consumption is not, suggesting that former procedure monitors redox activity of particle-bound metals, whereas the latter the redox activity of mostly organic species, as it is discussed in this paper.

The electron transfer is monitored by the rate at which DTT is consumed under a standardized set of conditions and the rate is proportional to the concentration of the catalytically active redox-active species in the sample. In brief, the Biosampler PM samples of known mass are incubated at 37°C with DTT (100 mM) in 0.1 M potassium phosphate buffer at pH 7.4 (1 mL total volume) for times varying from 0 to 30 minutes, and the reaction quenched at preset times by addition of 10% trichloroacetic acid. An aliquot of the quenched mixture is then transferred to a tube containing Tris HCl (0.4 M, pH 8.9), EDTA (20 mM) and 5,5'-dithiobis-2-nitrobenzoic acid (DTNB, 0.25 mM). The concentration of the remaining DTT is determined from the concentration of the 5-mercapto-2-nitrobenzoic acid formed by its absorption at 412 nm. The DTT consumed is determined from the difference between the mercaptobenzoate formed by the blank and that formed by the sample. The data collected at the multiple time points is used to determine the rate of DTT consumption, which is normalized to the quantity of PM used in the incubation mixture.

## Abbreviations

DTT dithiothreitol

EC elemental carbon

OC organic carbon

PAH polycyclic aromatic hydrocarbons

PM particulate matter

PTFE polytetrafluoroethylene

ROS reactive oxygen species

VACES versatile aerosol concentration enrichment system

## Competing interests

The author(s) declare that they have no competing interests.

## Authors' contributions

LN collected and processed the data, performed the statistical analysis and evaluated the results. JRF participated in the data evaluation and interpretation. AKC overviewed the DTT tests and reported the results of the analysis. CS conceived the study, and participated in its design and coordination and helped to draft the manuscript. All authors read and approved the final manuscript.
